# Supplementary material for: Enzymatic Synthesis of Unnatural Ginsenosides Using a Promiscuous UDP-Glucosyltransferase from Bacillus subtilis
Source: Molecules. 2018 Oct 28;23(11):2797. doi: 10.3390/molecules23112797 (PMC6278262; doi:10.3390/molecules23112797)

Supplementary Information

Table S1 ^{1}H-NMR (600 MHz) and ^{13}C-NMR (150 MHz) spectral data for product **1** in pyridine-d_5.

C	δ_C	δ_H (mult, J in Hz)
1	39.7	0.97 (1H, m); 1.54 (1H, m)
2	27.2	2.01 (1H, m); 2.03 (1H, m)
3	89.6	3.38 (1H, dd, J = 13.2, 4.8 Hz)
4	40.7	
5	61.5	1.32 (1H, s)
6	74.8	4.37 (1H, m)
7	45.9	2.23 (1H, m); 2.37 (1H, m)
8	41.7	
9	50.1	1.46 (1H, m)
10	39.5	
11	31.5	1.46 (1H, m); 2.24 (1H, m)
12	70.7	4.16 (1H, m)
13	49.7	2.16 (1H, s)
14	52.0	
15	31.3	0.76 (1H, m); 1.33 (1H, d, J = 10.8 Hz)
16	27.0	1.27 (1H, m); 1.94 (1H, m)
17	52.2	2.54 (1H, m)
18	17.9	1.13 (3H, s)
19	18.0	1.00 (3H, s)
20	83.8	
21	22.9	1.59 (3H, s)
22	36.6	1.56 (1H, m); 2.53 (1H, m)
23	23.8	2.24 (1H, m); 2.54 (1H, m)
24	126.4	5.24 (1H, m)
25	131.6	
26	26.3	1.61 (3H, s)
27	18.5	1.61 (3H, s)
28	32.1	2.12 (3H, s)
29	18.3	1.40 (3H, s)
30	17.8	0.91 (3H, s)
6-*O*-Glc-1′	102.4	5.19 (1H, d, J = 7.2 Hz)
2′	78.9	4.16 (1H, m)
3′	79.9	4.22 (1H, m)
4′	73.0	4.08 (1H, m)
5′	78.9	3.94 (1H, m)
6′	63.7	4.32 (1H, m); 4.49 (1H, m)
2′-*O*-Rham-1″	102.3	6.49 (1H, s)

2″	72.8	4.70 (1H, dd, *J* = 9.6, 3.6 Hz)
3″	72.5	4.61 (1H, m)
4″	75.7	4.34 (1H, m)
5″	70.2	4.80 (1H, m)
6″	19.4	1.80 (3H, d, *J* = 6.0 Hz)
20-*O*-Glc-1‴	98.8	5.18 (1H, d, *J* = 7.2 Hz)
2‴	75.7	4.01 (1H, m)
3‴	79.4	4.20 (1H, m)
4‴	72.2	4.07 (1H, m)
5‴	78.8	3.92 (1H, m)
6‴	63.5	4.24 (1H, m); 4.59 (1H, m)
3-*O*-Glc-1⁗	107.6	4.97 (overlapped)
2⁗	76.5	4.70 (1H, m)
3⁗	79.8	4.23 (1H, m)
4⁗	73.0	4.15 (1H, m)
5⁗	79.1	3.95 (1H, m)
6⁗	64.4	4.35 (1H, m); 4.59 (1H, m)

Table S2 ^{1}H-NMR (500 MHz) and ^{13}C-NMR (125 MHz) spectral data for product **2** in pyridine-d_5.

C	δ_C	δ_H (mult, J in Hz)
1	39.3	0.58 (1H, m); 1.53 (1H, m)
2	27.5	1.78 (1H, m); 1.84 (1H, m)
3	90.2	3.39 (1H, dd, J = 11.5, 4.0 Hz)
4	40.7	
5	61.9	1.23 (1H, m)
6	80.7	4.25 (1H, m)
7	44.5	1.95 (1H, td, J = 13.5, 3.0 Hz); 2.33 (1H, m)
8	41.1	
9	50.0	1.26 (1H, m)
10	40.8	
11	31.7	1.28 (1H, m); 2.25 (1H, m)
12	79.1	3.81 (1H, m)
13	46.7	2.02 (1H, m)
14	52.5	
15	28.0	1.16 (1H, t, J = 10.0 Hz); 1.29 (1H, m)
16	27.2	1.21 (1H, m); 1.73 (1H, m)
17	54.4	2.31 (1H, m)
18	17.6	0.85 (3H, s)
19	17.8	0.75 (3H, s)
20	73.3	
21	26.7	1.31 (3H, s)
22	36.8	1.62 (1H, m); 2.23 (1H, m)
23	23.3	1.29 (1H, m); 2.29 (1H, m)
24	126.9	5.35 (1H, t, J = 7.0 Hz)
25	131.0	
26	26.2	1.65 (3H, s)
27	18.1	1.64 (3H, s)
28	31.5	2.16 (3H, s)
29	17.3	1.55 (3H, s)
30	17.2	0.65 (3H, s)
6-*O*-Glc-1′	104.5	5.88 (1H, d, J = 7.5 Hz)
2′	80.1	4.37 (1H, m)
3′	80.4	4.23 (1H, m)
4′	72.0	4.13 (1H, m)
5′	78.8	3.92 (1H, m)
6′	63.0	4.30 (1H, m); 4.53 (1H, m)
2′-*O*-Glc-1″	103.8	4.78 (1H, d, J = 7.5 Hz)
2″	76.3	4.49 (1H, m)
3″	78.9	4.29 (1H, m)

4″	72.1	4.21 (1H, m)
5″	78.6	4.12 (1H, m)
6″	63.1	4.37 (1H, m) ; 4.38 (1H, m)
3-*O*-Glc-1‴	107.8	5.03 (1H, d, *J* = 7.5 Hz)
2‴	76.4	4.47 (1H, m)
3‴	78.9	4.25 (1H, m)
4‴	71.7	4.17 (1H, m)
5‴	78.3	4.05 (1H, m)
6‴	63.1	4.37 (1H, m); 4.55 (1H, m)
12-*O*-Glc-1⁗	100.6	5.28 (1H, d, *J* = 7.5 Hz)
2⁗	75.7	4.48 (1H, m)
3⁗	78.8	4.27 (1H, m)
4⁗	71.7	4.18 (1H, m)
5⁗	77.6	4.11 (1H, m)
6⁗	63.5	4.37 (1H, m); 4.66 (1H, m)

Table S3 ^{1}H-NMR (600 MHz) and ^{13}C-NMR (150 MHz) spectral data for product **3** in pyridine-d_5.

C	δ_C	δ_H (mult, J in Hz)
1	39.4	0.81(1H, m); 1.71 (1H, m)
2	27.4	2.01 (1H, m); 2.03 (1H, m)
3	90.3	3.43 (1H, dd, J = 12.0, 4.2 Hz)
4	40.8	
5	62.0	1.37 (1H, m)
6	80.3	4.26 (1H, m)
7	44.9	1.96 (1H, t, J = 12.0 Hz); 2.35 (1H, dd, J = 12.0, 3.6 Hz)
8	41.4	
9	50.4	1.50 (1H, m)
10	39.4	
11	31.8	1.47 (1H, m); 2.31 (1H, m)
12	71.4	3.84 (1H, m)
13	48.7	2.05 (1H, m)
14	52.1	
15	31.6	1.24 (1H, t, J = 10.2 Hz); 1.53 (1H, m)
16	26.8	1.35 (1H, m); 1.88 (1H, m)
17	55.2	2.38 (1H, m)
18	17.8	1.11 (3H, s)
19	17.9	0.90 (3H, s)
20	73.4	
21	27.2	1.42 (3H, s)
22	36.2	1.85 (1H, m); 2.33 (1H, m)
23	23.4	2.33 (1H, m); 2.62 (1H, m)
24	126.7	5.34 (1H, t, J = 6.6 Hz)
25	131.2	
26	26.2	1.67 (3H, s)
27	18.1	1.64 (3H, s)
28	32.4	2.16 (3H, s)
29	17.4	1.56 (3H, s)
30	17.2	0.85 (3H, s)
6-*O*-Glc-1′	104.0	5.86 (1H, d, J = 7.8 Hz)
2′	80.3	4.38 (1H, m)
3′	80.9	4.29 (1H, m)
4′	71.8	4.07 (1H, m)
5′	78.7	3.91 (1H, m)
6′	63.2	4.23 (1H, m); 4.51 (1H, m)
2′-*O*-Glc-1″	104.5	4.84 (1H, d, J = 7.2 Hz)
2″	76.4	4.49 (1H, m)
3″	78.8	4.38 (1H, m)

4″	72.1	4.17 (1H, m)
5″	78.3	4.01 (1H, m)
6″	63.2	4.28 (1H, m) ; 4.60 (1H, m)
3-*O*-Glc-1‴	107.7	5.02 (1H, d, *J* = 7.8 Hz)
2‴	76.4	4.43 (1H, m)
3‴	78.9	4.30 (1H, m)
4‴	72.2	4.14 (1H, m)
5‴	78.6	3.92 (1H, m)
6‴	63.5	4.24 (1H, m); 4.51 (1H, m)

Table S4 ^{1}H-NMR (500 MHz) and ^{13}C-NMR (125 MHz) spectral data for product **4** in methanol-d_4.

C	δ_C	δ_H (mult, J in Hz)
1	39.9	1.14 (1H, s); 1.70 (1H, m)
2	27.7	1.88 (1H, m); 1.97 (1H, m)
3	90.9	3.22 (1H, m)
4	40.9	
5	62.0	1.30 (1H, m)
6	80.8	4.06 (1H, td, J = 10.5, 3.0 Hz)
7	45.2	1.90 (1H, m); 3.06 (1H, m)
8	41.8	
9	50.7	1.41 (1H, m)
10	40.2	
11	31.7	1.41 (1H, m); 2.05 (1H, m)
12	79.4	3.23 (1H, m)
13	46.9	1.80 (1H, m)
14	53.1	
15	28.6	1.13 (1H, m); 1.54 (1H, m)
16	26.8	1.26 (1H, m); 1.76 (1H, m)
17	55.1	2.09 (1H, m)
18	17.6	1.04 (3H, s)
19	17.9	0.97 (3H, s)
20	74.8	
21	26.1	1.08 (3H, s)
22	36.7	1.58 (1H, m); 1.80 (1H, m)
23	23.3	2.07 (1H, m); 3.08 (1H, m)
24	126.3	5.11 (1H, t, J = 7.0 Hz)
25	131.9	
26	25.9	1.60 (3H, s)
27	17.8	1.36 (3H, s)
28	31.1	1.65 (3H, s)
29	16.8	1.09 (3H, s)
30	17.3	0.93 (3H, s)
6-*O*-Glc-1′	105.6	4.31 (1H, d, J = 8.0 Hz)
2′	75.7	3.17 (1H, td, J = 8.5, 2.5 Hz)
3′	79.0	3.58 (1H, m)
4′	71.7	330 (1H, m)
5′	78.0	3.11 (1H, m)
6′	62.9	3.77 (1H, s); 3.87 (1H, td, J = 10.5, 5.0 Hz)
3-*O*-Glc-1″	107.0	4.29 (1H, d, J = 8.0 Hz)
2″	75.5	3.17 (1H, td, J = 8.5, 2.5 Hz)
3″	78.4	3.30 (1H, m)
4″	71.7	3.24 (1H, m)

5″	77.7	3.06 (1H, m)
6″	62.8	3.63 (1H, m); 3.79 (1H, m)
12-*O*-Glc-1‴	100.6	4.47 (1H, d, *J* = 8.0 Hz)
2‴	75.1	3.17 (1H, td, *J* = 8.5, 2.5 Hz)
3‴	78.3	3.33 (1H, m)
4‴	71.0	3.25 (1H, m)
5‴	77.6	3.08 (1H, m)
6‴	62.4	3.64 (1H, m); 3.82 (1H, m)

Table S5 ^{1}H-NMR (500 MHz) and ^{13}C-NMR (125 MHz) spectral data for product **5** in methanol-d_4.

C	δ_C	δ_H (mult, J in Hz)
1	40.1	1.41 (1H, s); 1.71 (1H, m)
2	27.4	1.75 (1H, m); 1.87 (1H, m)
3	90.9	3.27 (1H, m)
4	40.9	
5	62.0	1.14 (1H, m)
6	80.8	4.09 (1H, td, J = 10.5, 2.5 Hz)
7	45.4	1.85 (1H, m); 3.12 (1H, dd, J = 12.0, 4.5 Hz)
8	41.8	
9	50.9	1.53 (1H, m)
10	40.0	
11	32.0	1.50 (1H, s); 2.02 (1H, m)
12	71.7	3.89 (1H, m)
13	49.6	1.97 (1H, m)
14	52.5	
15	31.2	1.22 (1H, m); 1.55 (1H, s)
16	26.8	1.29 (1H, m); 1.73 (1H, m)
17	55.1	2.15 (1H, m)
18	17.6	1.07 (3H, s)
19	17.7	1.00 (3H, s)
20	74.4	
21	26.5	1.09 (3H, s)
22	36.3	1.64 (1H, m); 2.00 (1H, m)
23	23.3	2.05 (1H, m); 3.26 (1H, m)
24	126.2	5.14 (1H, t, J = 7.0 Hz)
25	132.0	
26	25.9	1.62 (3H, s)
27	17.9	1.40 (3H, s)
28	31.9	1.68 (3H, s)
29	16.8	1.15 (3H, s)
30	17.0	0.94 (3H, s)
6-*O*-Glc-1′	105.6	4.34 (1H, d, J = 7.5 Hz)
2′	75.8	3.65 (1H, td, J = 11.5, 5.5 Hz)
3′	79.1	3.35 (1H, m)
4′	72.1	334 (1H, m)
5′	77.7	3.20 (1H, td, J = 7.5, 2.0 Hz)
6′	62.9	3.83 (1H, t, J = 12.0 Hz); 4.63 (1H, s)
3-*O*-Glc-1″	107.0	4.32(1H, d, J = 7.5 Hz)
2″	75.3	3.64 (1H, td, J = 11.5, 5.5 Hz)
3″	78.4	3.55 (1H, m)
4″	71.7	3.33 (1H, m)

5″	77.7	3.20 (1H, td, *J* = 7.5, 2.0 Hz)
6″	62.8	3.83 (1H, t, *J* = 12.0 Hz); 4.10 (1H, td, *J* = 10.5, 2.5 Hz)

Table S6 ^{1}H-NMR (600 MHz) and ^{13}C-NMR (150 MHz) spectral data for product **6** in pyridine-d_5.

C	δ_C	δ_H (mult, J in Hz)
1	41.0	0.97 (1H, m); 1.77 (1H, m)
2	27.2	1.94 (1H, m); 1.96 (1H, m)
3	90.1	3.45 (1H, dd, J = 11.4, 4.2 Hz)
4	41.6	
5	63.5	1.29 (1H, s)
6	75.7	4.49 (1H, t, J = 13.2 Hz)
7	49.8	2.03 (1H, m); 2.34 (1H, m)
8	45.3	
9	52.0	1.44 (1H, m)
10	39.7	
11	31.5	1.44 (1H, m); 2.20 (1H, m)
12	70.7	4.00 (1H, m)
13	50.4	2.00 (1H, m)
14	52.0	
15	31.2	0.81 (1H, m); 1.35 (1H, d, J = 10.2 Hz)
16	26.9	1.28 (1H, m); 1.90 (1H, m)
17	61.9	2.53 (1H, m)
18	17.8	1.10 (3H, s)
19	18.0	0.91 (3H, s)
20	83.8	
21	22.9	1.60 (3H, m)
22	36.7	1.80 (1H, m); 2.39 (1H, m)
23	23.8	2.25 (1H, m); 2.50 (1H, m)
24	126.5	5.25 (1H, t, J = 7.2 Hz)
25	131.5	
26	26.3	1.60 (3H, m)
27	18.3	1.61 (3H, m)
28	31.7	2.15 (3H, s)
29	18.1	1.48 (3H, s)
30	17.8	0.84 (3H, s)
6-*O*-Glc-1′	103.9	4.91 (1H, d, J = 7.8 Hz)
2′	79.3	4.18 (1H, m)
3′	80.4	4.15 (1H, m)
4′	72.1	4.13 (1H, m)
5′	78.9	3.95 (1H, m)
6′	64.4	4.24 (1H, m); 4.35 (1H, m)
2′-*O*-Rham-1″	105.8	5.67 (1H, m)
2″	72.5	4.49 (1H, t, J = 13.2 Hz)
3″	71.7	4.42 (1H, m)
4″	74.6	4.31 (1H, m)

5″	66.0	4.60 (1H, m)
20-*O*-Glc-1‴	98.8	5.19 (1H, d, *J* = 7.8 Hz)
2‴	76.4	3.84 (1H, m)
3‴	79.9	4.15 (1H, m)
4‴	67.9	4.07 (1H, m)
5‴	78.8	3.65 (1H, t, *J* = 11.4 Hz)
6‴	63.7	4.23 (1H, m); 4.34 (1H, m)
3-*O*-Glc-1⁗	107.6	5.02 (1H, d, *J* = 7.8 Hz)
2⁗	78.7	4.22 (1H, m)
3⁗	81.7	4.16 (1H, m)
4⁗	72.3	4.14 (1H, m)
5⁗	79.4	3.01 (1H, m)
6⁗	65.4	4.25 (1H, m); 4.40 (1H, m)

Table S7 The HPLC conditions of scale-up preparation for products **1-6**.

products	conditions	t_R (min)
**1**	0 min, 20% ACN; 20 min, 35% ACN; 31 min, 100% ACN; 40min, 100% ACN	7.87 (**1**)
**2** and **3**	0 min, 20% ACN; 30 min, 40% ACN; 31 min, 100% ACN; 40min, 100% ACN	7.28 (**2**); 12.88 (**3**)
**4** and **5**	0 min, 20% ACN; 20 min, 40% ACN; 31 min, 100% ACN; 40min, 100% ACN	8.21 (**4**); 14.19 (**5**)
**6**	0 min, 20% ACN; 30 min, 35% ACN; 31 min, 100% ACN; 40min, 100% ACN	7.01 (**6**)

Figure S1. The ^{1}H NMR (A), ^{13}C NMR (B), HMBC (C) and HSQC (D) spectra of product **1** in pyridine-d_5.

A

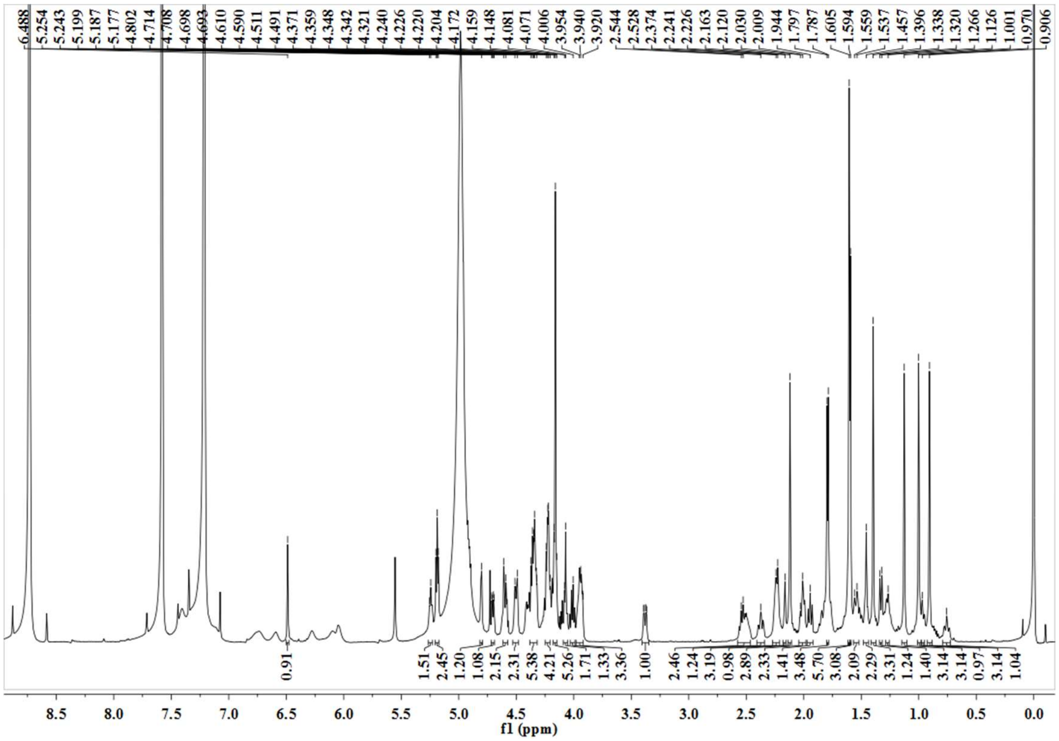

B

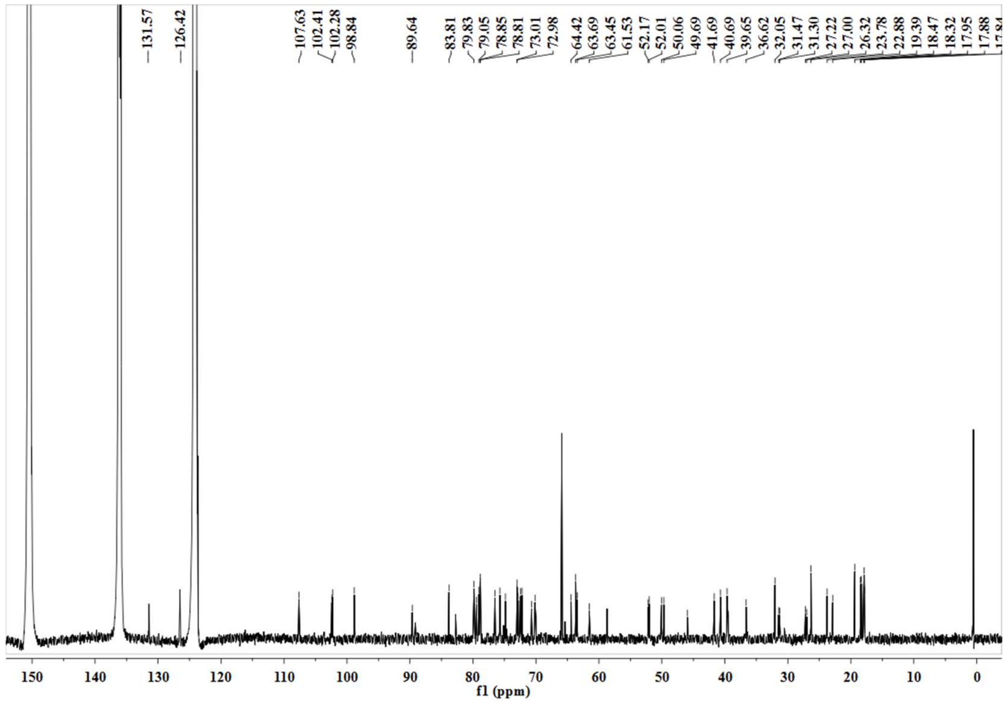

C

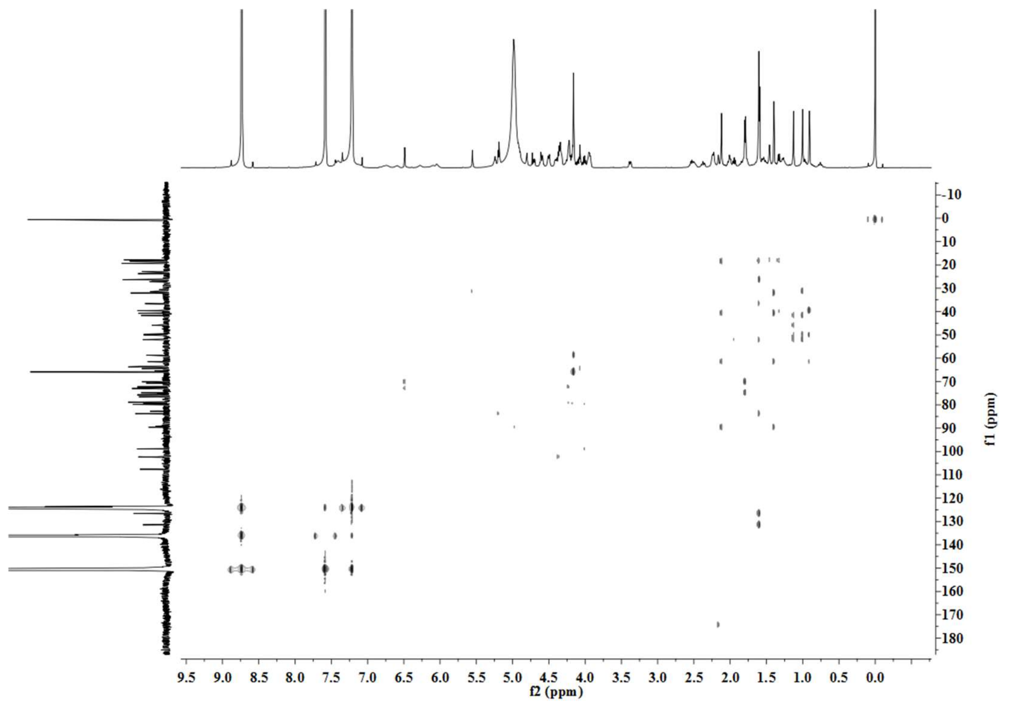

D

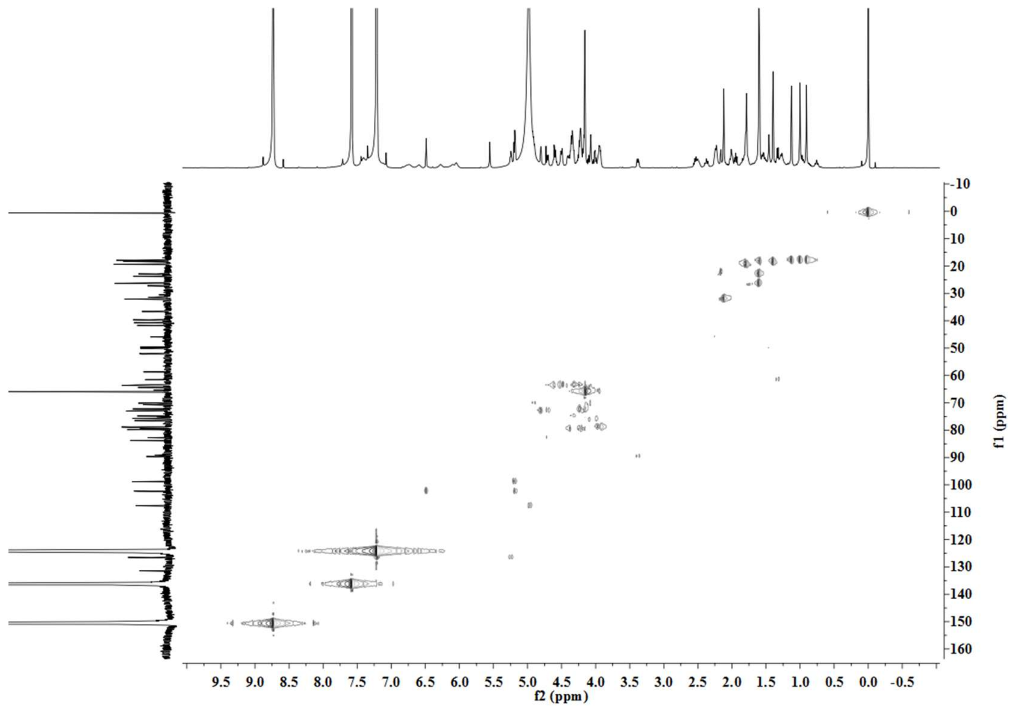

Figure S2. The ^{1}H NMR (A), ^{13}C NMR (B), HMBC (C) and HSQC (D) spectra of product **2** in pyridine-d_5.

A

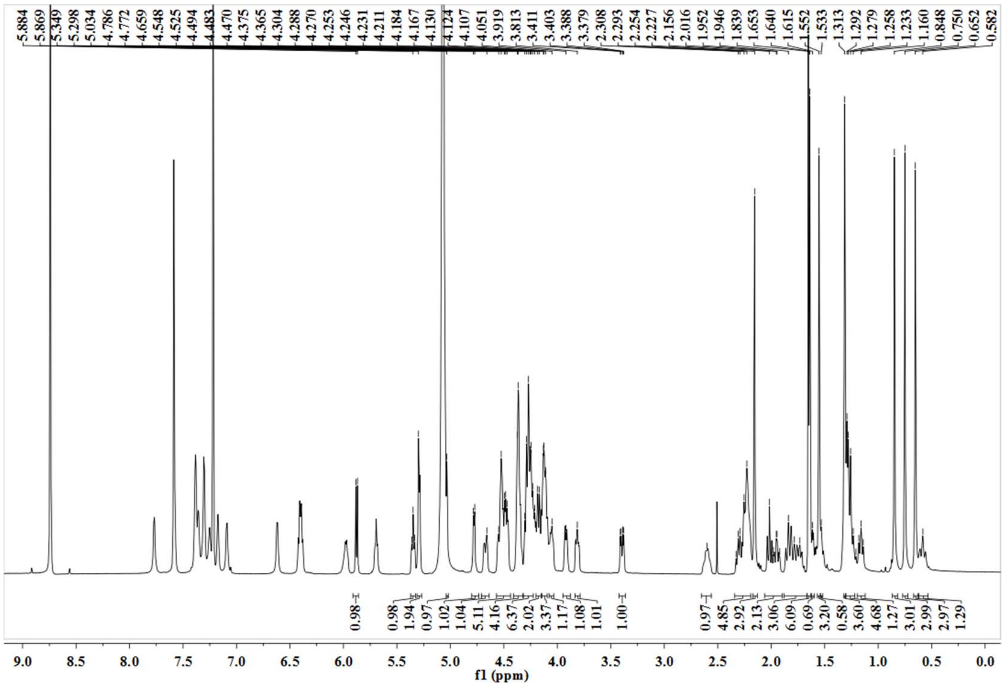

B

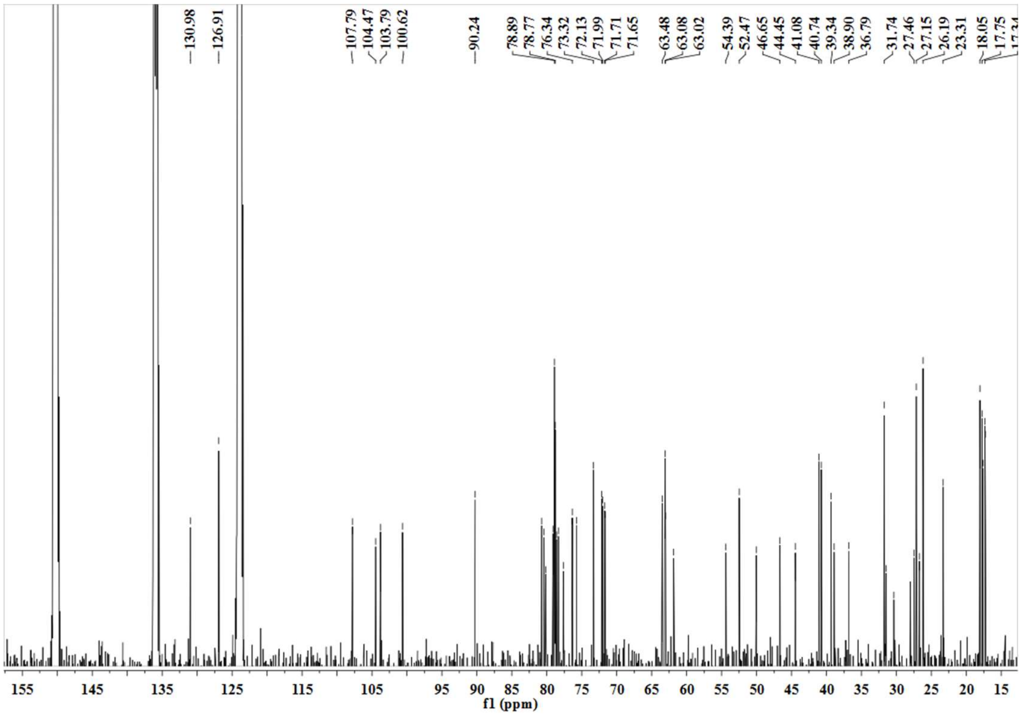

C

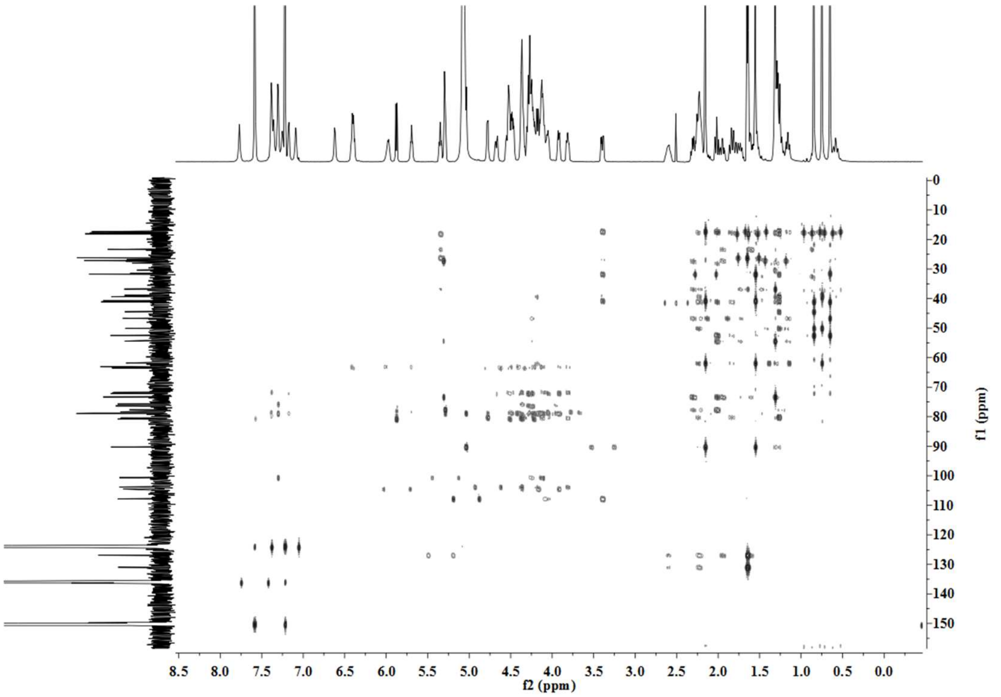

D

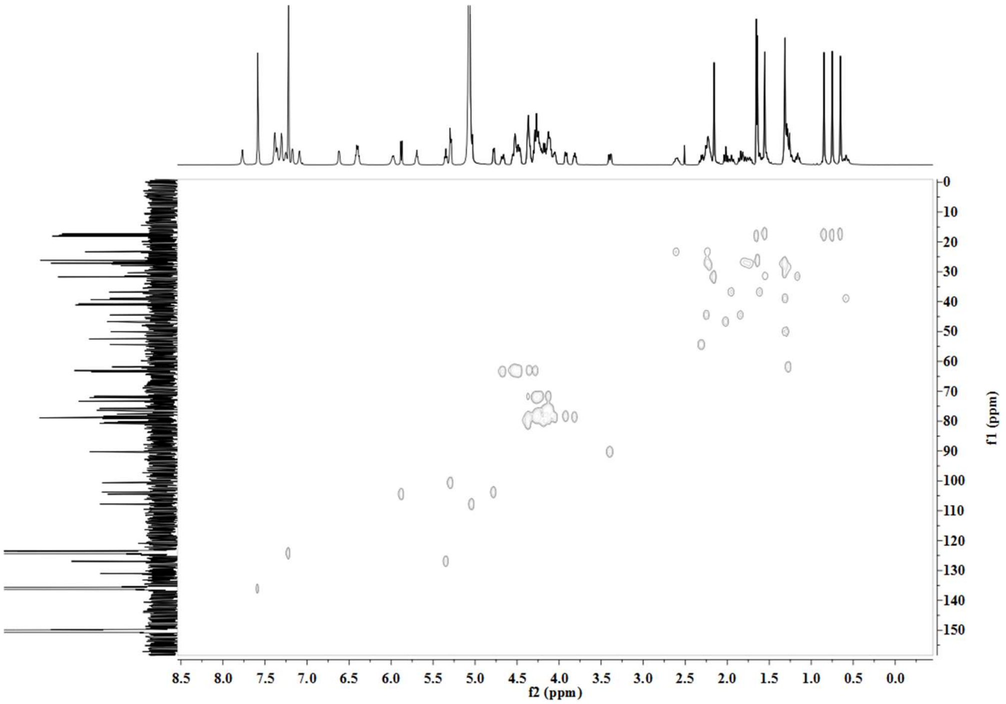

Figure S3. The ^{1}H NMR (A), ^{13}C NMR (B), HMBC (C) and HSQC (D) spectra of product **3** in pyridine-d_5.

A

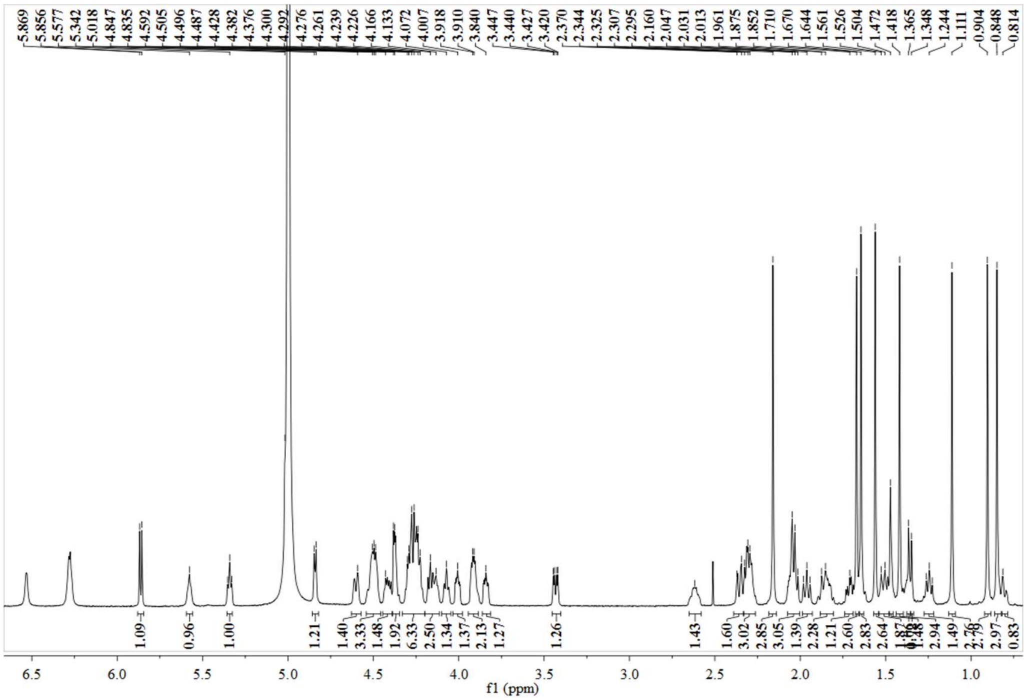

B

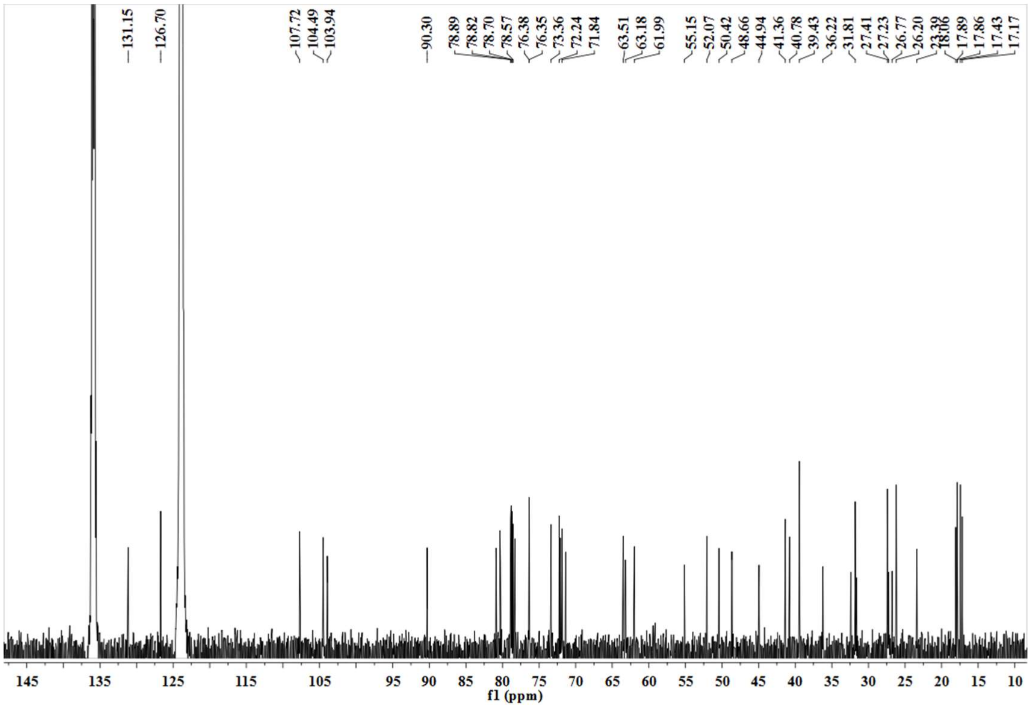

C

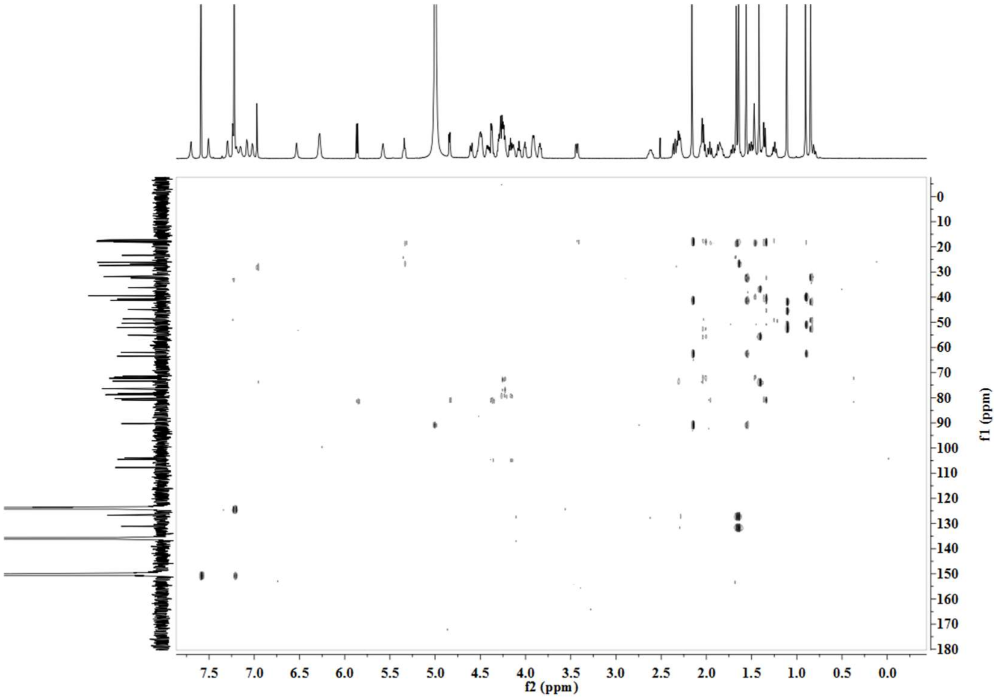

D

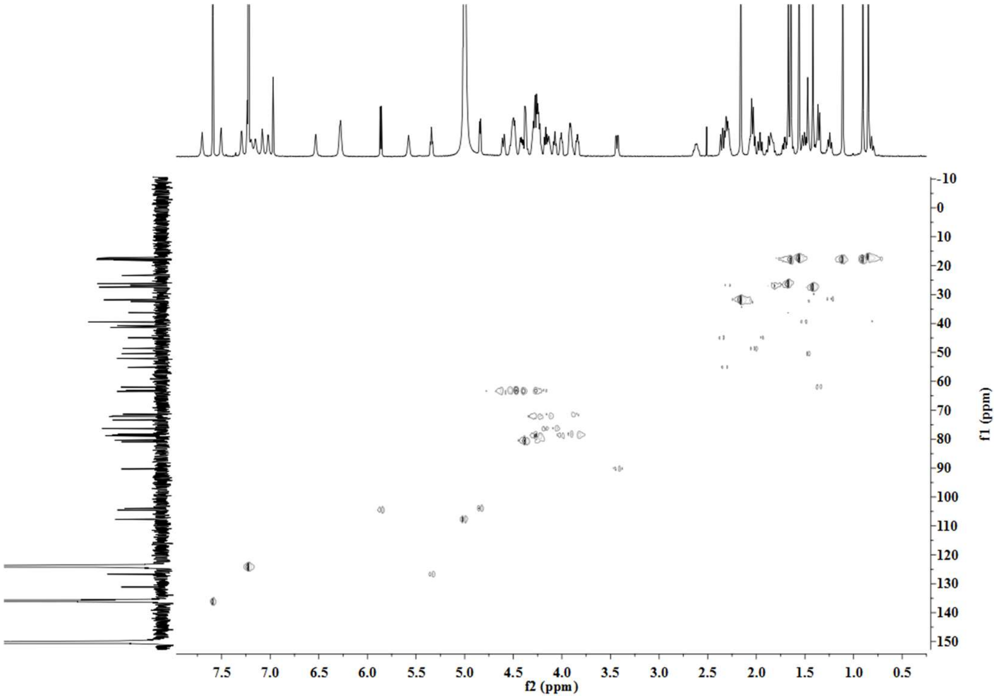

Figure S4. The ^{1}H NMR (A), ^{13}C NMR (B), HMBC (C) and HSQC (D) spectra of product **4** in methanol-d_4.

A

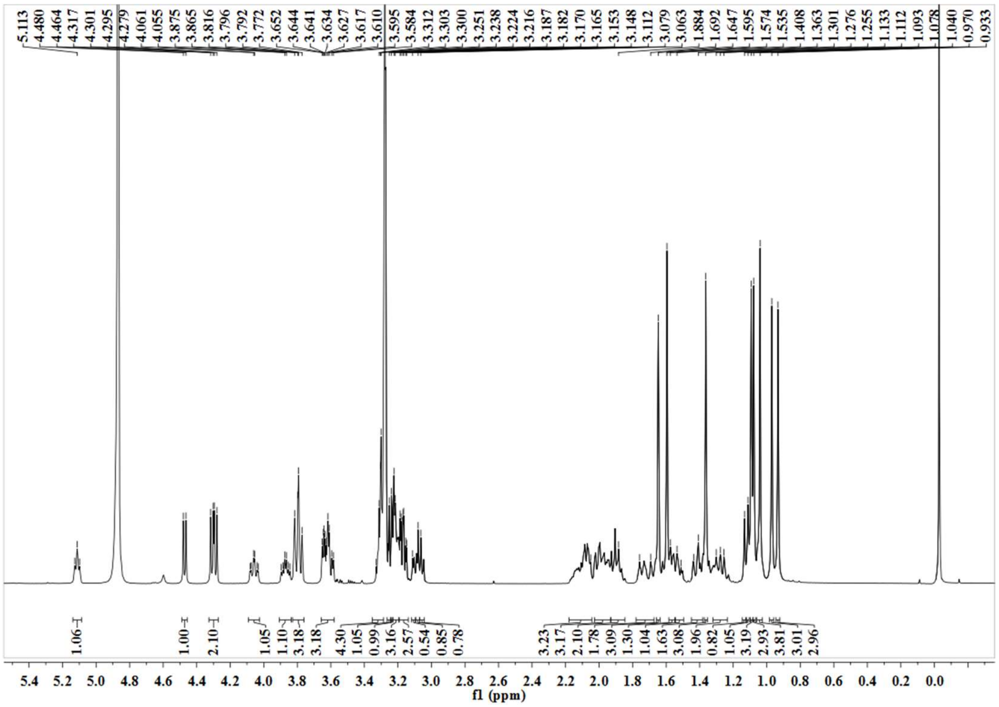

B

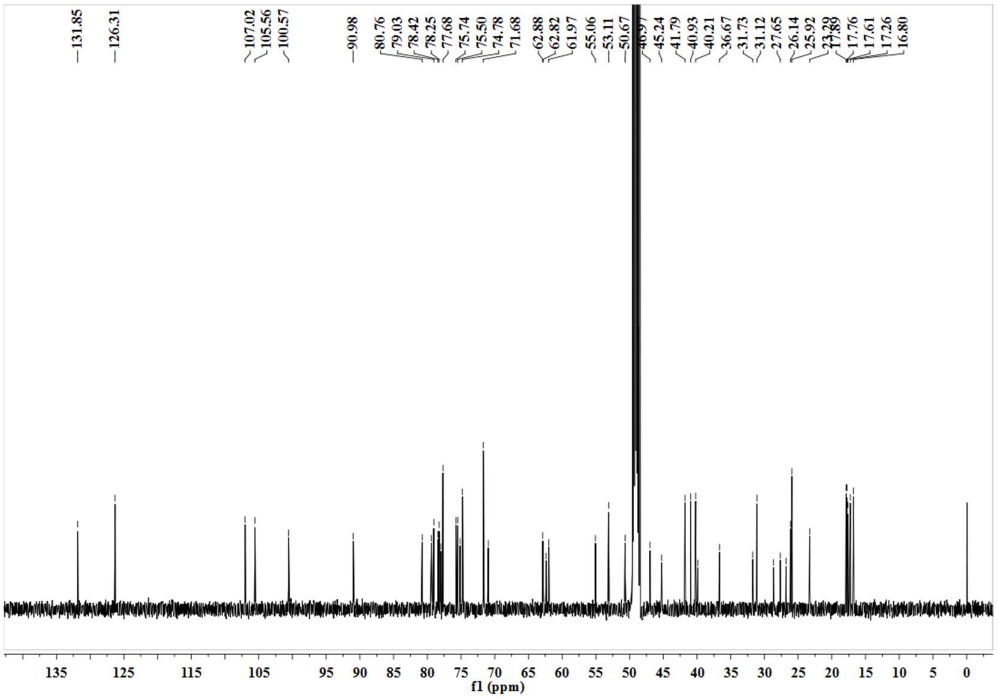

C

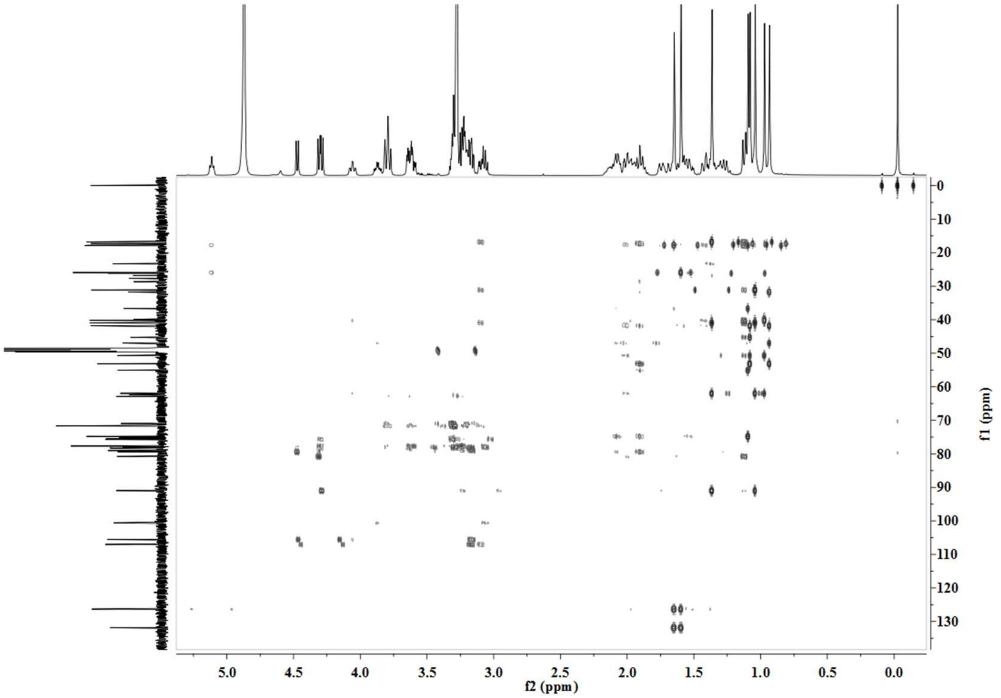

D

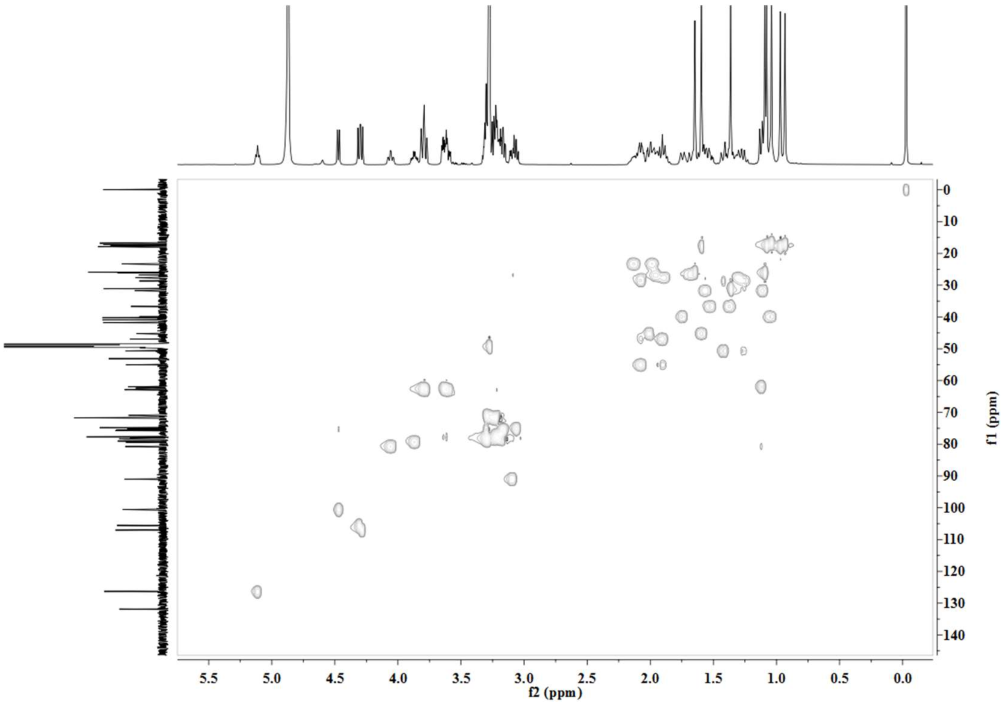

Figure S5. The ^{1}H NMR (A) and ^{13}C NMR (B) spectra of product **5** in methanol-d_4.

A

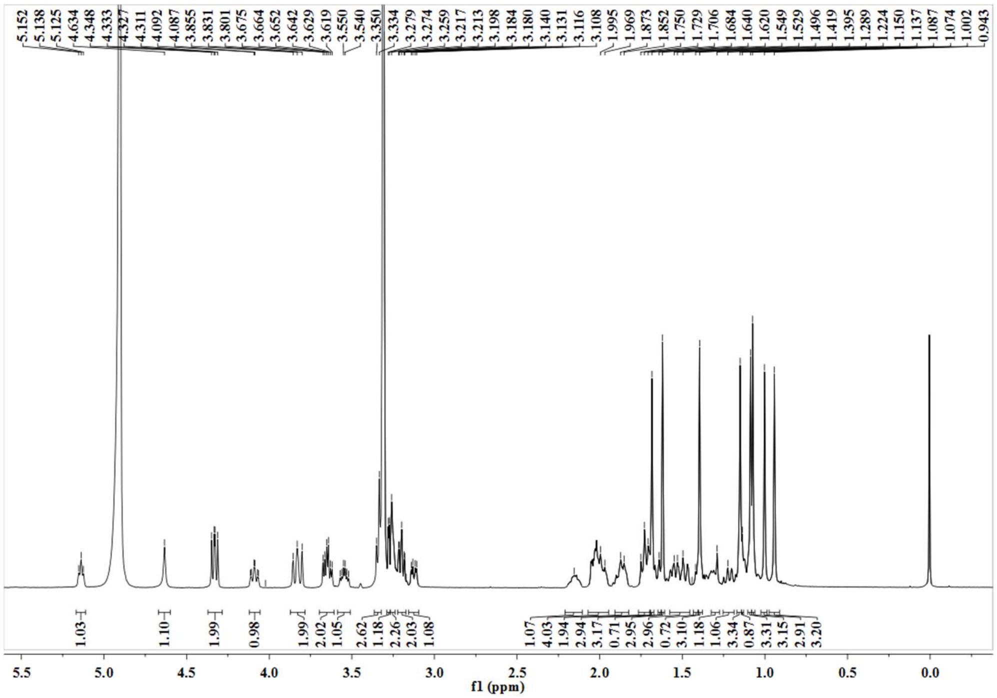

B

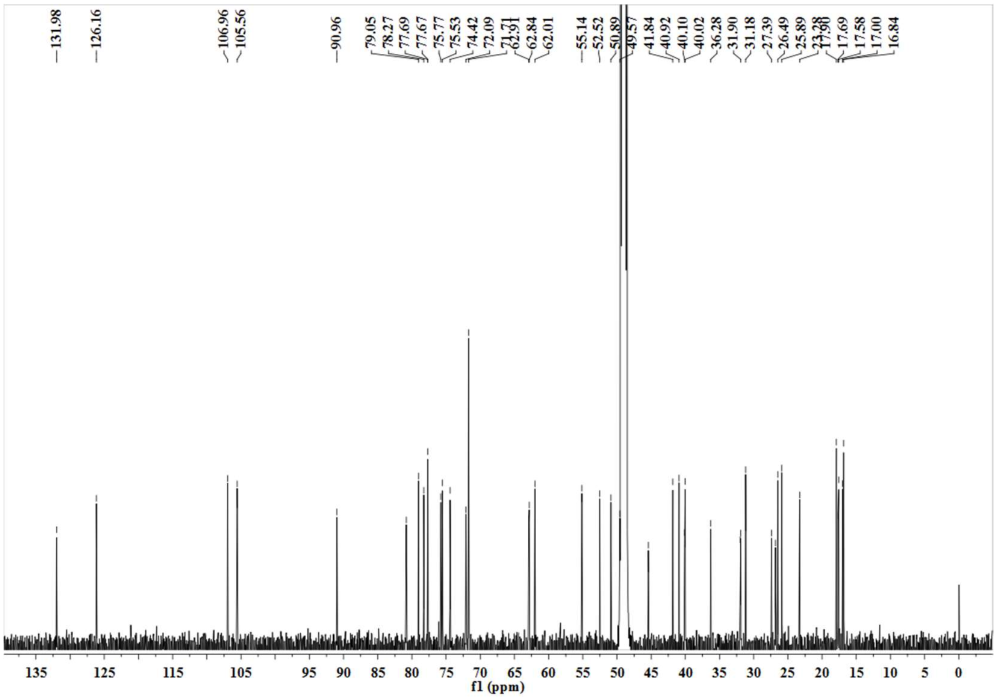

Figure S6. The ^{1}H NMR (A), ^{13}C NMR (B), HMBC (C) and HSQC (D) spectra of product **6** in pyridine-d_5.

A

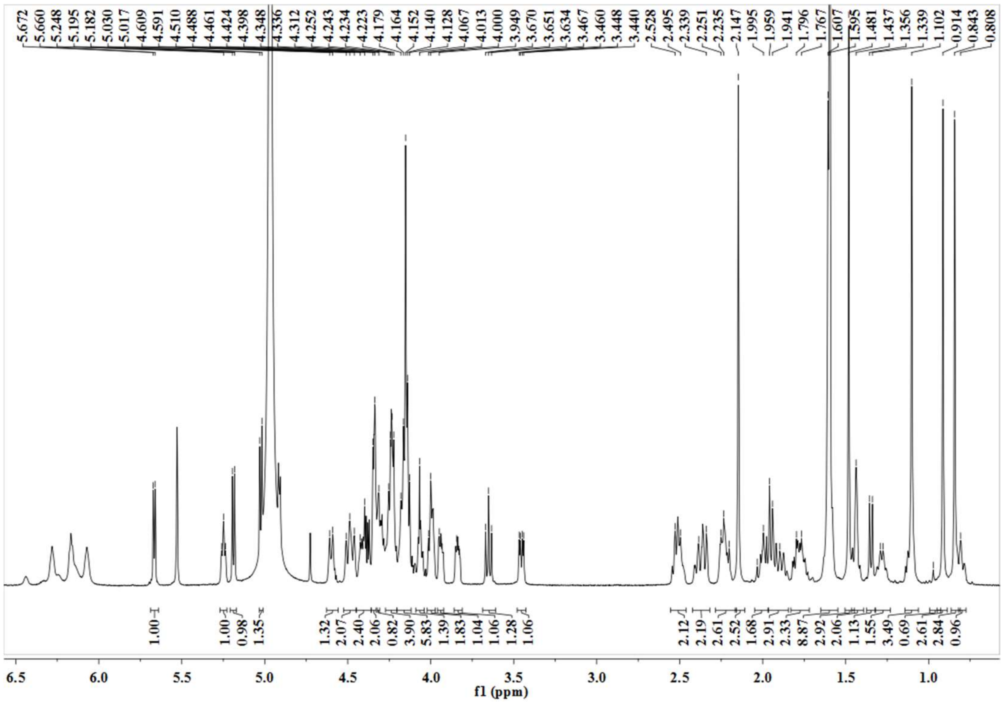

B

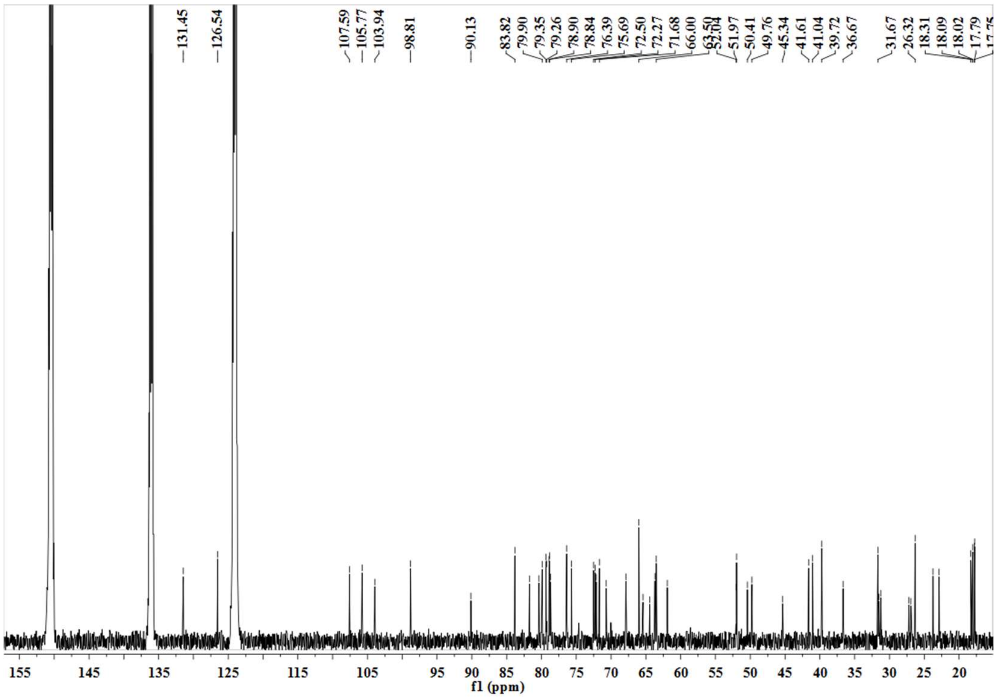

C

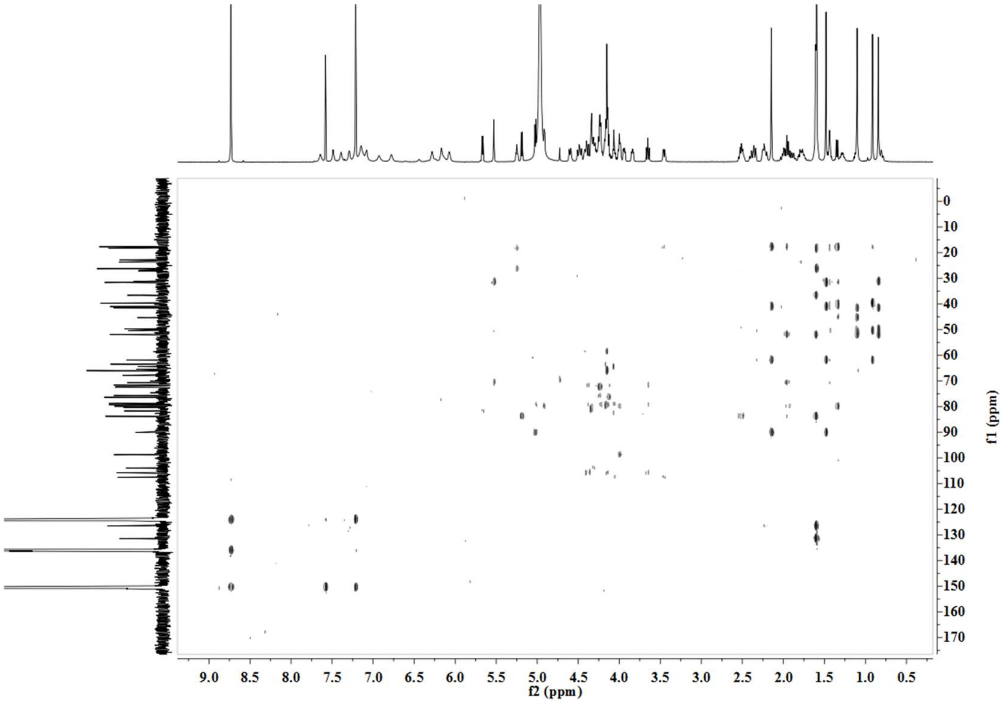

D

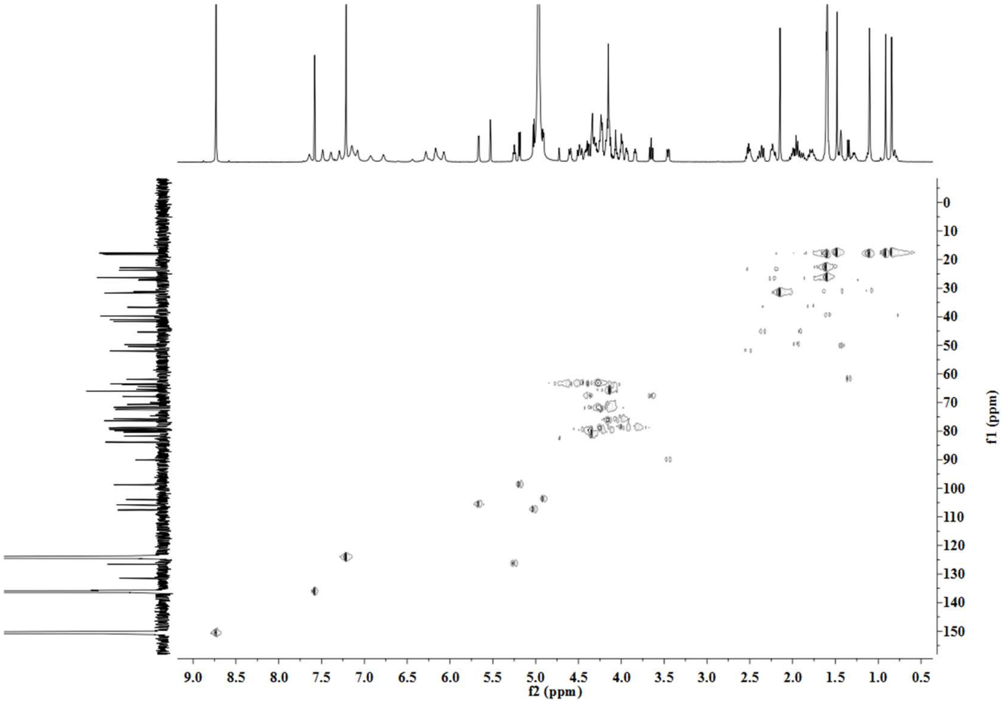

Supplement: Supplementary file 1 [file molecules-23-02797-s001.pdf]
